# Natural Language Processing in a Clinical Decision Support System for the Identification of Venous Thromboembolism: Algorithm Development and Validation

**DOI:** 10.2196/43153

**Published:** 2023-04-24

**Authors:** Zhi-Geng Jin, Hui Zhang, Mei-Hui Tai, Ying Yang, Yuan Yao, Yu-Tao Guo

**Affiliations:** 1 Department of Pulmonary Vascular and Thrombotic Disease Sixth Medical Center of Chinese People's Liberation Army General Hospital Beijing China; 2 Chinese People's Liberation Army Medical School Beijing China; 3 Quality Management Division Sixth Medical Center of Chinese People's Liberation Army General Hospital Beijing China; 4 Institute for Hospital Management Research Chinese People's Liberation Army General Hospital Beijing China

**Keywords:** venous thromboembolism, deep vein thrombosis, pulmonary embolism, natural language processing, electronic health record

## Abstract

**Background:**

It remains unknown whether capturing data from electronic health records (EHRs) using natural language processing (NLP) can improve venous thromboembolism (VTE) detection in different clinical settings.

**Objective:**

The aim of this study was to validate the NLP algorithm in a clinical decision support system for VTE risk assessment and integrated care (DeVTEcare) to identify VTEs from EHRs.

**Methods:**

All inpatients aged ≥18 years in the Sixth Medical Center of the Chinese People's Liberation Army General Hospital from January 1 to December 31, 2021, were included as the validation cohort. The sensitivity, specificity, positive and negative likelihood ratios (LR+ and LR–, respectively), area under the receiver operating characteristic curve (AUC), and F1-scores along with their 95% CIs were used to analyze the performance of the NLP tool, with manual review of medical records as the reference standard for detecting deep vein thrombosis (DVT) and pulmonary embolism (PE). The primary end point was the performance of the NLP approach embedded into the EHR for VTE identification. The secondary end points were the performances to identify VTE among different hospital departments with different VTE risks. Subgroup analyses were performed among age, sex, and the study season.

**Results:**

Among 30,152 patients (median age 56 [IQR 41-67] years; 14,247/30,152, 47.3% females), the prevalence of VTE, PE, and DVT was 2.1% (626/30,152), 0.6% (177/30,152), and 1.8% (532/30,152), respectively. The sensitivity, specificity, LR+, LR–, AUC, and F1-score of NLP-facilitated VTE detection were 89.9% (95% CI 87.3%-92.2%), 99.8% (95% CI 99.8%-99.9%), 483 (95% CI 370-629), 0.10 (95% CI 0.08-0.13), 0.95 (95% CI 0.94-0.96), and 0.90 (95% CI 0.90-0.91), respectively. Among departments of surgery, internal medicine, and intensive care units, the highest specificity (100% vs 99.7% vs 98.8%, respectively), LR+ (3202 vs 321 vs 77, respectively), and F1-score (0.95 vs 0.89 vs 0.92, respectively) were in the surgery department (all *P*<.001). Among low, intermediate, and high VTE risks in hospital departments, the low-risk department had the highest AUC (1.00 vs 0.94 vs 0.96, respectively) and F1-score (0.97 vs 0.90 vs 0.90, respectively) as well as the lowest LR– (0.00 vs 0.13 vs 0.08, respectively) (DeLong test for AUC; all *P*<.001). Subgroup analysis of the age, sex, and season demonstrated consistently good performance of VTE detection with >87% sensitivity and specificity and >89% AUC and F1-score. The NLP algorithm performed better among patients aged ≤65 years than among those aged >65 years (F1-score 0.93 vs 0.89, respectively; *P*<.001).

**Conclusions:**

The NLP algorithm in our DeVTEcare identified VTE well across different clinical settings, especially in patients in surgery units, departments with low-risk VTE, and patients aged ≤65 years. This algorithm can help to inform accurate in-hospital VTE rates and enhance risk-classified VTE integrated care in future research.

## Introduction

Venous thromboembolism (VTE), which includes deep vein thrombosis (DVT) and pulmonary embolism (PE), is a leading cause of death and disability worldwide [1,2]. Studies have shown that the prevalence of VTE has substantially increased worldwide [1-4]. Up to 60% of VTE cases occur during or after hospitalization, making it a leading preventable cause of hospital death [5]. The detection of VTE among hospitalized patients informs decision-making surrounding VTE risk and thrombosis risk mitigation. However, doctors and nurses may not be aware of the risk of VTE, which in turn leads to lack of adherence with the clinical practice guidelines for patients with VTE, given the complex clinical setting [6,7]. To ensure that best practices are reliably delivered, some quality and safety indicators for VTE prevention and management have been adopted as measures of hospital performance and are utilized in several pay-for-performance programs [8,9]. Thus, accurate and timely identification of VTE from hospital clinical and administrative databases is crucial for assessing hospital care quality and for further enhancing improvements in care.

Although manual medical record review is the gold standard for VTE event investigation, it is a time-consuming, labor-intensive, and costly process. The International Classiﬁcation of Diseases diagnosis codes are commonly used to monitor VTE incidence at each hospital, but they are unreliable to establish ground truth since they are prone to coding bias and have variable reported accuracy in identifying VTE [10-12]. Moreover, they are only available after discharge. The growing use of electronic health records (EHRs) has made it possible for health care providers to acquire an invaluable source of information for research and analysis. Recently, the natural language processing (NLP) method has been developed to embed into EHRs to flag the adverse outcomes [13,14]. These technologies, which use corpus processing and automatic learning models from extracted EHR data, hold promise for reliably and efficiently identifying patients with VTE [15].

Previous studies on the application of NLP tools for detecting VTE cases mainly utilized imaging reports, and the reported positive predictive values (PPVs) ranged from 54% to 100% [10,11,16-24]. Nonetheless, besides imaging, more available data, including clinical text, laboratory values, and vital signs in EHRs, can be utilized for NLP to improve VTE detection. In addition, VTE prophylaxis is hospital-level management, and NLP-facilitated VTE detection should adapt to different clinical settings and help clinicians pay attention to VTE in the early stage.

In this study, we describe an NLP algorithm in a clinical decision support system for VTE risk assessment and integrated care (DeVTEcare) that can timely recognize DVT and PE from different types of EHR data after their occurrence and we validated its performance of VTE detection in different clinical settings.

## Methods

### Study Design and Population

This retrospective observational study was conducted in the Sixth Medical Center of the Chinese People's Liberation Army General Hospital, which is a tertiary hospital that integrates medical care, teaching, and research. All inpatients aged ≥18 years admitted between January 1 and December 31, 2021, were included as the validation cohort. Hospitalized patients were excluded if they lacked diagnostic information or if their hospital stay was ≤24 hours. This study followed the Standards for Reporting Diagnostic accuracy studies (STARD) reporting guidelines (Multimedia Appendix 1) [25].

### Ethics Approval

This cohort study was approved by the medical ethics committee of the Sixth Medical Center of Chinese People's Liberation Army General Hospital (approval HZKY-PJ-2022-21). A waiver of informed consent was obtained because this was a retrospective data-only study. All data were deidentified.

### NLP Approach Development

The rule-based NLP approach in DeVTEcare was embedded into the EHR to timely identify VTE based on the clinical text (admission notes, medical progress notes, surgical notes, discharge summaries, etc) and imaging reports. When a patient was admitted, DeVTEcare automatically assessed and flagged VTE risk, and then, the guideline-recommended VTE prophylaxis and treatments were further proposed at 5 time points: within 24 hours after admission, within 24 hours before and after operation, within 24 hours after transferring, and at discharge. Moreover, VTE risk assessment was automatically evaluated daily at midnight.

**Figure 1:**
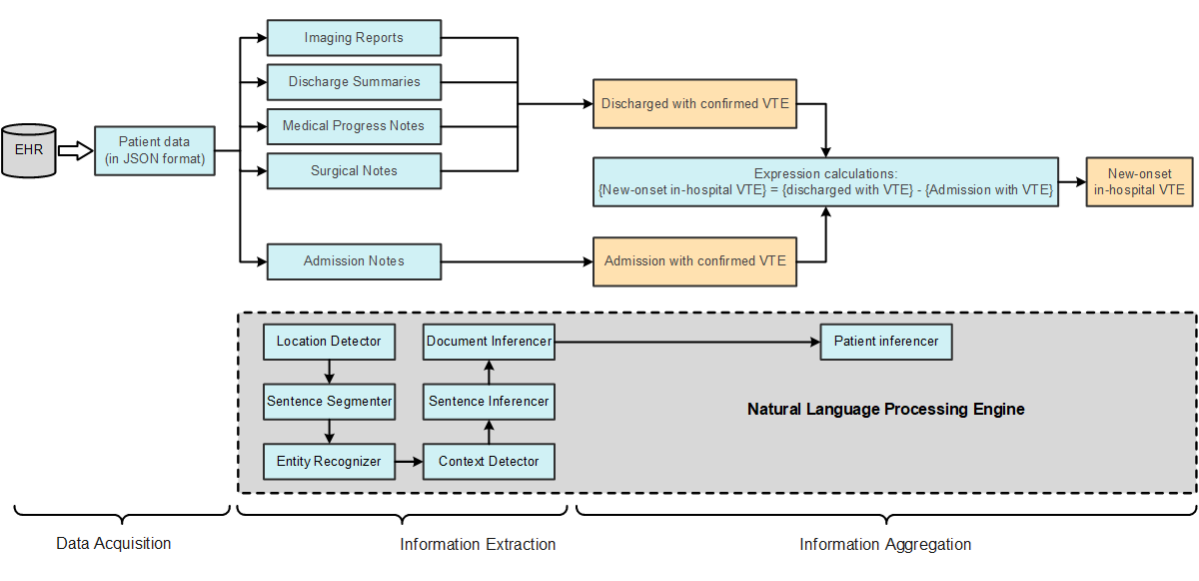
The natural language processing tool inferencing workflow in the clinical decision support system for venous thromboembolism risk assessment and integrated care. EHR: electronic health record; VTE: venous thromboembolism.

The NLP system detected VTE in 3 steps: data acquisition, information extraction, and information aggregation (Figure 1). This system employs a set of highly optimized and configurable NLP components that enable quick and easy clinical information extraction and aggregation (Table 1). All relevant full-text documents from patients were retrieved from the EHR. First, the data acquisition module encapsulated patient data into the JSON format. Second, in the information extraction process, the location of extraction was determined by the location detector. The information for the discharge confirmation of patients with VTE was extracted from the patient’s imaging report, discharge summaries, medical progress notes, and surgical notes, while the information for the admission confirmation of patients with VTE was extracted from the patient’s admission notes. Then, the sentence segmenter was used to break up the sentence, the entity recognizer was used to identify the target concept, and the context detector was used to find the target concept’s occurrence time and negation relationship. Next, the sentence inferencer was used to derive the sentence conclusion, and the document inferencer was then utilized to generate the document conclusion. Third, in the information aggregation process, the convergence of various documented findings yielded “discharged with VTE” and “admission with VTE,” which were then further computed by expressions to yield the results for new-onset in-hospital VTE.

**Table 1:** Components of the natural language processing tool in the clinical decision support system for venous thromboembolism risk assessment and integrated care.

Components	Functionality description
Location detector	Identify information extraction locations such as present medical history, admission notes, medical progress notes, surgical notes, discharge summaries, and imaging reports
Sentence segmenter	Detect sentence boundaries
Entity recognizer^a^	Identify target concepts such as deep vein thrombosis or pulmonary embolism
Context detector	Attach the context information as feature values to the corresponding target concepts such as present or historical, negated or affirmed
Sentence inferencer	Create sentence-level conclusions based on target concepts and corresponding context
Document inferencer	Create a document-level conclusion from the corresponding sentence-level conclusions
Patient inferencer^b^	Aggregate document-level conclusions to infer patient-level conclusions

^a^As an extension, the entity recognizer can not only extract named entity recognition from free text but also extract variables from structural data.

^b^For the patient inferencer part, we have expanded the syntax expression function to support arithmetic, logic, and set operations.

To achieve these objectives, a VTE domain knowledge base was developed. We used disease definitions from the Chinese Guidelines for the Prevention and Treatment of Thrombophilia [26], the National Clinical Version of Disease Classification and Coding 2.0 [27], and the corresponding Chinese translations from the Unified Medical Language System from the National Library of Medicine. We also searched medical websites for corresponding synonyms to enrich the ontology concepts [28]. The knowledge base was further improved by a research team involving 2 NLP programmers, 3 medical specialists in thrombosis, and 2 database administrators with a weekly working meeting. During the iterative training phase, a small proportion of real clinical cases was manually verified by the designated physicians on a weekly basis, the NLP programmer refined the recognition error of the rules iteratively for 8 weeks, and eventually, 200 randomly selected patients were tested. During the testing phase, additional 260 patients were randomly selected for testing by the physicians. After the test was complete, the NLP approach achieved 85.7% sensitivity and 93.5% specificity for detecting VTE. Then, the NLP programmer repeatedly refined the rules based on the test results and research team’s comments until all correctable faults were fixed. The keywords for the NLP algorithm are listed in Multimedia Appendix 2.

### Validation of the NLP Approach

The NLP approach for VTE detection was validated in different clinical settings and in departments with different VTE risk statuses, as follows.

1. We stratified 43 inpatient departments in our hospital into surgery, internal medicine, and intensive care units.
2. The number of VTE-related deaths was calculated between the years of 2011 and 2020.

The departments were further classified as low, intermediate, and high risks for VTE if there were <1, 1-5, >5 VTE-related deaths per year, respectively. The performance of the NLP approach was also evaluated with regard to the type of VTE, that is, PE or DVT. Manual review of medical records was taken as the reference standard for detecting PE and DVT. When there was a disagreement between NLP identification and the clinical expert’s review, an advanced artificial intelligence engineer and a cardiologist audited the event.

### Study End Points

The primary end point was the performance of the NLP approach in DeVTEcare embedded into the EHR for VTE identification. The secondary end points were the performances to identify VTE in different clinical settings and in departments with different VTE risk statuses.

### Clinical Events

The diagnosis of VTE was confirmed on ultrasound doppler, computed tomography, or computed tomography pulmonary angiogram. The VTEs were classified as (1) on-admission VTE: new-onset VTE admitted to the hospital and (2) in-hospital VTE: new-onset VTE during hospitalization but without VTE on admission. Since this study primarily focused on DVT and PE, thrombosis in the upper extremity, internal jugular, and superior or inferior vena cava were not considered valid VTE. Patients with prior VTE were also deemed invalid.

### Statistical Analyses

Continuous variables were expressed as median (IQR), while categorical variables were expressed as numbers and percentages. The sensitivity, specificity, PPV, negative predictive value (NPV), area under the receiver operating characteristic curve (AUC), and F1-score were used to evaluate the performance of the NLP approach for VTE detection, in relation to the type of VTE, different clinical settings, and in the low-, intermediate-, and high-risk departments. Subgroup analyses were performed among age (>65 years or ≤65 years), sex (female or male), and the study season (first quarter, second quarter, third quarter, fourth quarter). Given that PPV might be affected by the prevalence of disease, positive and negative likelihood ratios (LR+ and LR–, respectively) were used to characterize the clinical utility of the test. The LR– CIs were obtained using a bootstrap method with 100% sensitivity [29].

The chi-square test or Fisher exact test was applied for the comparisons of the categorical variables, if appropriate. DeLong test was used for comparing AUCs, and *Z* test was used for comparing the F1-scores. The relative diagnostic LR method was used for comparing the LRs of 2 diagnostic tests [30]. The Bonferroni method was used to adjust for multiple comparisons. The agreement to the reference test and the interreviewer agreement were also assessed using Cohen κ test. A 2-sided *P*<.01 was considered to be statistically signiﬁcant. All statistical analyses were carried out using the R software (version 4.2.1; R Core Team and the R Foundation for Statistical Computing).

## Results

### Characteristics of the Patients

**Figure 2:**
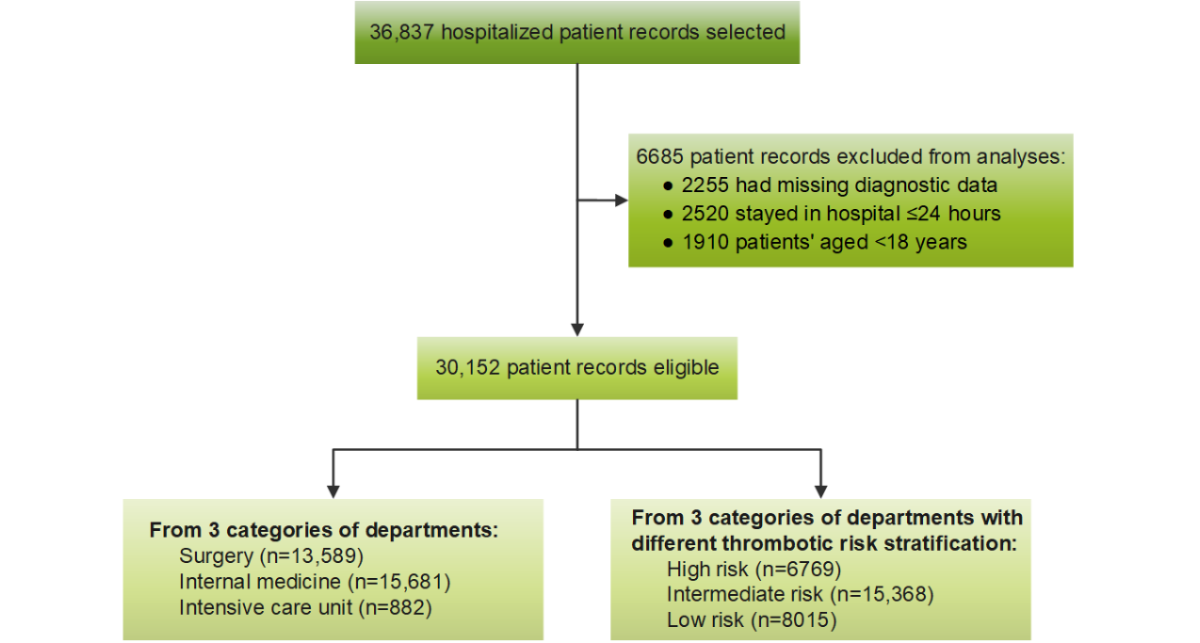
Flowchart of the patient analysis in this study. The low, intermediate, and high risks of venous thromboembolism were defined by departments having <1, 1-5, and >5 venous thromboembolism–related deaths per year, respectively.

A total of 30,152 patients were included from January 1 to December 31, 2021; the flow chart is shown in Figure 2. The median patient age was 56 (IQR 41-67) years, and 47.3% (14,247/30,152) of the patients were females. Half of the patients were from internal medicine departments with moderate risk of VTE. The enrolled patients were almost equally distributed throughout the 4 quarters of the study year. The median length of hospital stay was 8 (IQR 5-12) days. The incidence of VTE confirmed by the clinical experts was 2.1% (626/30,152), with PE at 0.6% (177/30,152) and DVT at 1.8% (532/30,152). The detailed incidence of VTE is shown in Table 2 and Multimedia Appendix 3. Overall, the prevalence of VTE increased with the increasing VTE risk in the departments (high risk: 219/6769, 3.2%; intermediate risk: 358/15,368, 2.3%; low risk: 49/8015, 0.6%; *P*<.001).

**Table 2:** Characteristics of the patients in this study.

Characteristics			Patients (N=30,152), n (%)		Venous thromboembolism events^a^ (n=626), n (%)		*P* value^b^
**Age (years)**							<.001
	>65	8859 (29.4)		409 (4.6)			
	≤65	21,293 (70.6)		217 (1)			
**Sex**							.43
	Female	14,247 (47.3)		286 (2)			
	Male	15,905 (52.7)		340 (2.1)			
**Department**							<.001
	Surgery	13,589 (45.1)		77 (0.6)			
	Internal medicine	15,681 (52)		421 (2.7)			
	Intensive care unit	882 (2.9)		128 (14.5)			
**Venous thromboembolism risk^c^**							<.001
	High	6769 (22.4)		219 (3.2)			
	Intermediate	15,368 (51)		358 (2.3)			
	Low	8015 (26.6)		49 (0.6)			
**Quarter of the study year**							<.001
	First quarter	5868 (19.5)		109 (1.9)			
	Second quarter	7616 (25.2)		128 (1.7)			
	Third quarter	8510 (28.2)		157 (1.8)			
	Fourth quarter	8158 (27.1)		232 (2.8)			

^a^Any venous thromboembolism event confirmed by clinical experts.

^b^*P* values for the comparison of venous thromboembolism events.

^c^The low, intermediate, and high venous thromboembolism risk was defined by departments having <1, 1-5, and >5 venous thromboembolism–related deaths per year, respectively.

### NLP Performance for VTE Detection

**Figure 3:**
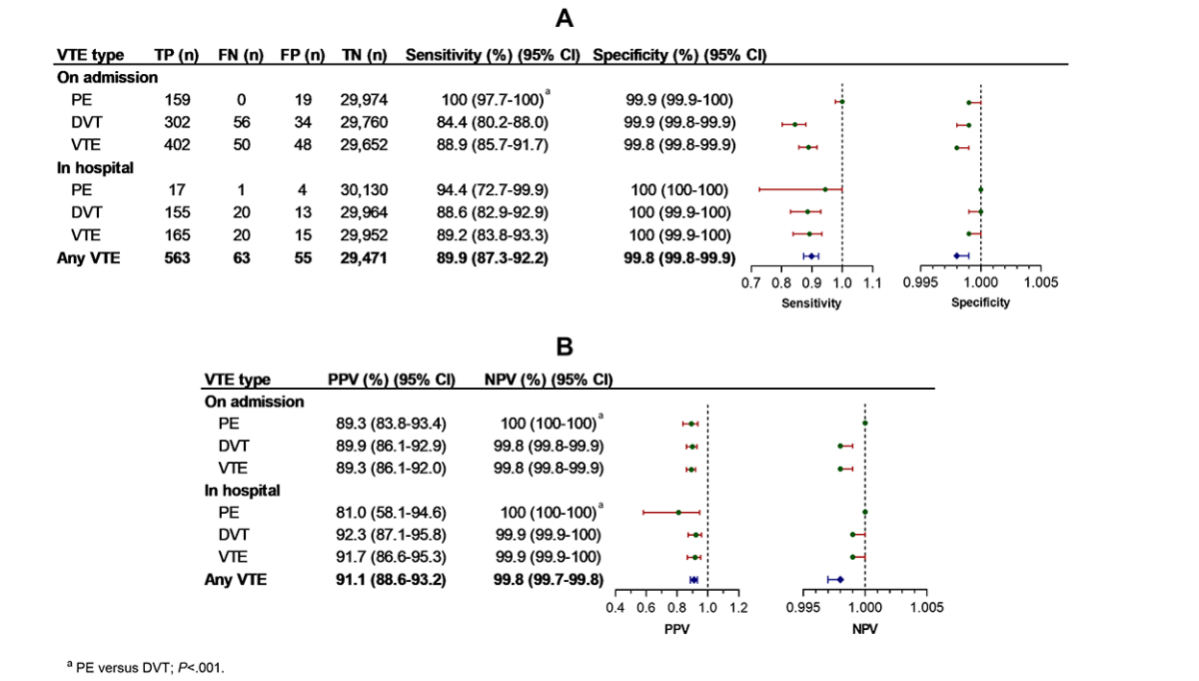
Performance of the clinical decision support system for venous thromboembolism risk assessment and integrated care for identifying venous thromboembolism. (A) Sensitivity and specificity. (B) Positive and negative predictive values. DVT: deep vein thrombosis; FN: false negative; FP: false positive; NPV: negative predictive value; PE: pulmonary embolism; PPV: positive predictive value; TN: true negative; TP: true positive; VTE: venous thromboembolism.

In DeVTEcare, of the 626 events, 563 (89.9%) VTEs were detected by the NLP approach, achieving a sensitivity of 89.9%, specificity of 99.8%, PPV of 91.1%, NPV of 99.8%, LR+ of 483, LR– of 0.1, AUC of 0.95, and F1-score of 0.9. The NLP approach detected the on-admission and in-hospital VTE, with sensitivity ranging from 84.4% to 100%, specificity from 99.8% to 100%, PPV from 81% to 92.3%, NPV from 99.8% to 100%, LR+ from 550 to 7115, LR– from 0 to 0.16, AUC from 0.92 to 1.00, and F1-score from 0.87 to 0.94 (Table 3, Table 4, and Figure 3). However, NLP performed better in detecting PE than DVT for on-admission VTE (sensitivity 100% vs 84.4%, NPV 100% vs 99.8%, LR+ 1579 vs 739, LR– 0 vs 0.16, AUC 1.00 vs 0.92, F1-score 0.94 vs 0.87, respectively; DeLong test for AUC; all *P<*.01), while much higher NPV (100% vs 99.9%, respectively; *P<*.001) and lower F1-score (0.87 vs 0.90, respectively; *P<*.001) were observed for in-hospital PE detection. No statistically significant differences in the other diagnostic parameters between PE and DVT for either on-admission or in-hospital detection were found (all *P*>.01) (Table 3, Table 4, and Figure 3). DeVTEcare and the expert’s review agreed well for any type of VTE identification, with κ values ranging from 0.87 to 0.94 (Table 4). Interreviewer agreement for the diagnosis of VTE showed κ values of 0.90-1.00.

**Table 3:** Likelihood ratios of the clinical decision support system for venous thromboembolism risk assessment and integrated care for identifying venous thromboembolism.

Venous thromboembolism type			Positive likelihood ratio (95% CI)		Negative likelihood ratio (95% CI)
**On-admission** **venous thromboembolism**					
	Pulmonary embolism	1579 (1007-2474)^a^		0.00 (0.00-0.02)^b^	
	Deep vein thrombosis	739 (527-1037)		0.16 (0.12-0.20)	
	Venous thromboembolism	550 (414-731)		0.11 (0.08-0.14)	
**In-hospital** **venous thromboembolism**					
	Pulmonary embolism	7115 (2653-19,077)		0.06 (0.01-0.37)	
	Deep vein thrombosis	2042 (1183-3526)		0.11 (0.08-0.17)	
	Venous thromboembolism	1782 (1072-2962)		0.11 (0.07-0.16)	
	Any venous thromboembolism	483 (370-629)		0.10 (0.08-0.13)	

^a^Pulmonary embolism versus deep vein thrombosis; *P=*.008.

^b^Pulmonary embolism versus deep vein thrombosis; *P<*.001.

**Table 4:** Diagnostic statistics of the clinical decision support system for venous thromboembolism risk assessment and integrated care for identifying venous thromboembolism.

Venous thromboembolism type			Area under the curve^a^ (95% CI)		F1-score^b^ (95% CI)		κ (95% CI)
**On-admission** **venous thromboembolism**							
	Pulmonary embolism	1.00 (1.00-1.00)^c^		0.94 (0.93-0.95)^c^		0.94 (0.92-0.97)	
	Deep vein thrombosis	0.92 (0.90-0.94)		0.87 (0.86-0.88)		0.87 (0.84-0.90)	
	Venous thromboembolism	0.94 (0.93-0.96)		0.89 (0.88-0.90)		0.89 (0.87-0.91)	
**In-hospital** **venous thromboembolism**							
	Pulmonary embolism	0.97 (0.92-1.00)		0.87 (0.83-0.91)		0.87 (0.76-0.98)	
	Deep vein thrombosis	0.94 (0.92-0.97)		0.90 (0.89-0.92)^c^		0.90 (0.87-0.94)	
	Venous thromboembolism	0.95 (0.92-0.97)		0.90 (0.89-0.91)		0.90 (0.87-0.93)	
	Any venous thromboembolism	0.95 (0.94-0.96)		0.90 (0.90-0.91)		0.90 (0.89-0.92)	

^a^DeLong test was used for comparing areas under the curve.

^b^*Z* test was used for comparing F1-scores.

^c^Pulmonary embolism versus deep vein thrombosis; *P<*.001.

### NLP Performance in Different Settings and VTE Risk

**Figure 4:**
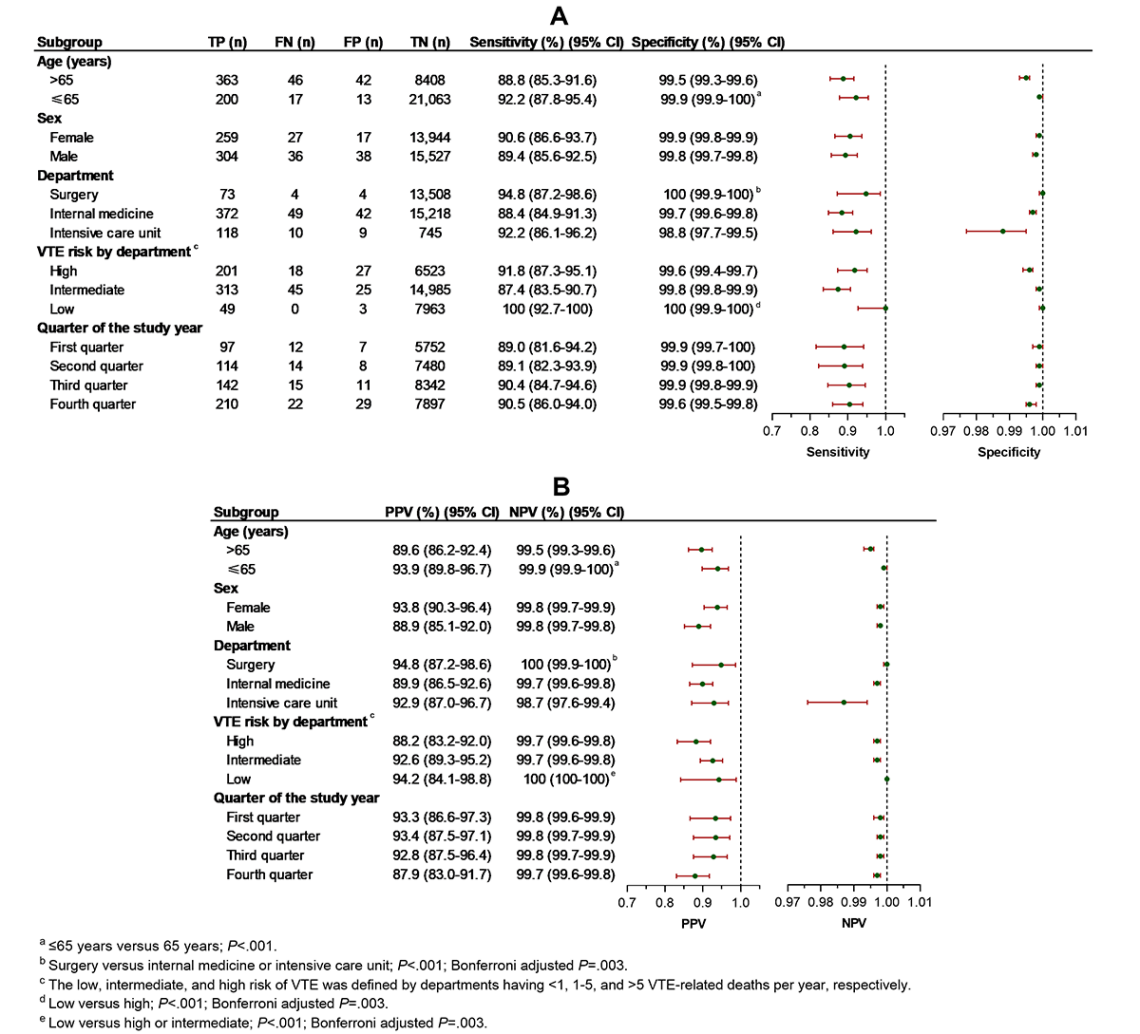
Diagnostic statistics of the clinical decision support system for venous thromboembolism risk assessment and integrated care for identifying any venous thromboembolism according to different settings. (A) Sensitivity and specificity. (B) Positive and negative predictive values. FN: false negative; FP: false positive; NPV: negative predictive value; PPV: positive predictive value; TN: true negative; TP: true positive; VTE: venous thromboembolism.

Among the departments of surgery, internal medicine, and intensive care units, the highest specificity (100% vs 99.7% vs 98.8%, respectively), NPV (100% vs 99.7% vs 98.7%, respectively), LR+ (3202 vs 321 vs 77, respectively), and F1-score (0.95 vs 0.89 vs 0.92, respectively) of the NLP approach for VTE detection were in the surgery department (all *P*<.001; Bonferroni adjusted *P*=.003). No statistically significant differences were found in sensitivity, PPV, LR–, and AUC (all *P*>.01; Bonferroni adjusted *P*=.003) (Table 5, Table 6, and Figure 4).

The NLP algorithm consistently detected VTE in low, intermediate, and high-risk departments with good sensitivity and specificity (sensitivity 100%, 87.4%, and 91.8%, respectively; specificity 100%, 99.8%, and 99.6%, respectively), although the difference of specificity between low- and high-risk departments was statistically significant (*P*<.001; Bonferroni adjusted *P*=.003). Among low-, intermediate-, and high-risk departments, the low-risk department had the highest AUC (1.00 vs 0.94 vs 0.96, respectively), F1-score (0.97 vs 0.90 vs 0.90, respectively), NPV (100% vs 99.7% vs 99.7%, respectively), and the lowest LR– (0.00 vs 0.13 vs 0.08, respectively) (DeLong test for AUC, all *P*<.001; Bonferroni adjusted *P*=.003). The highest LR+ was associated with the low-risk department as well (2655 vs 525 vs 223, respectively), but a statistically significant difference existed only between the low- and high-risk departments (*P*<.001; Bonferroni adjusted *P*=.003) (Table 5, Table 6, and Figure 4). The error analysis of DeVTEcare in detecting VTE is shown in Table 7.

**Table 5:** Likelihood ratios of the clinical decision support system for venous thromboembolism risk assessment and integrated care for identifying any venous thromboembolism according to different settings.

Setting			Positive likelihood ratio (95% CI)		Negative likelihood ratio (95% CI)
**Age (years)**					
	>65	178 (132-242)		0.11 (0.09-0.15)	
	≤65	1494 (866-2576)^a^		0.08 (0.05-0.12)	
**Sex**					
	Female	744 (462-1198)		0.09 (0.07-0.13)	
	Male	366 (266-504)		0.11 (0.08-0.14)	
**Department**					
	Surgery	3202 (1200-8543)^b^		0.05 (0.02-0.13)	
	Internal medicine	321 (237-435)		0.12 (0.09-0.15)	
	Intensive care unit	77 (40-148)		0.08 (0.04-0.14)	
**Venous thromboembolism risk by department^c^**					
	High	223 (152-325)		0.08 (0.05-0.13)	
	Intermediate	525 (354-778)		0.13 (0.10-0.16)	
	Low	2655 (856-8231)^d^		0.00 (0.00-0.06)	
**Quarter of the study year**					
	First quarter	732 (348-1539)		0.11 (0.06-0.19)	
	Second quarter	834 (416-1671)		0.11 (0.07-0.18)	
	Third quarter	687 (380-1242)		0.10 (0.06-0.15)	
	Fourth quarter	247 (171-357)		0.10 (0.06-0.14)	

^a^≤65 years versus >65 years; *P<*.001.

^b^Surgery versus internal medicine or intensive care unit; *P<*.001; Bonferroni adjusted *P*=.003.

^c^The low, intermediate, and high risk of venous thromboembolism was defined by departments having <1, 1-5, and >5 venous thromoboembolism–related deaths per year, respectively.

^d^Low versus high; *P<*.001; Bonferroni adjusted *P*=.003.

**Table 6:** Diagnostic statistics of the clinical decision support system for venous thromboembolism risk assessment and integrated care for identifying any venous thromboembolism according to different settings.

Setting			Area under the curve^a^ (95% CI)		F1-score^b^ (95% CI)	κ (95% CI)
**Age (years)**						
	>65	0.94 (0.93-0.96)		0.89 (0.88-0.90)		0.92 (0.88-0.95)
	≤65	0.96 (0.94-0.98)		0.93 (0.92-0.94)^c^		0.98 (0.96-0.99)
**Sex**						
	Female	0.95 (0.93-0.97)		0.92 (0.91-0.93)^d^		0.92 (0.90-0.94)
	Male	0.95 (0.93-0.96)		0.89 (0.88-0.90)		0.89 (0.87-0.91)
**Department**						
	Surgery	0.97 (0.95-1.00)		0.95 (0.94-0.96)^e^		0.95 (0.93-0.96)
	Internal medicine	0.94 (0.92-0.96)		0.89 (0.88-0.90)		0.89 (0.87-0.91)
	Intensive care unit	0.95 (0.93-0.98)		0.92 (0.91-0.94)		0.91 (0.90-0.93)
**Venous thromboembolism risk by department^f^**						
	High	0.96 (0.94-0.97)		0.90 (0.89-0.91)		0.90 (0.88-0.91)
	Intermediate	0.94 (0.92-0.95)		0.90 (0.89-0.91)		0.90 (0.88-0.91)
	Low	1.00 (1.00-1.00)^g^		0.97 (0.96-0.98)^g^		0.97 (0.96-0.98)
**Quarter of the study year**						
	First quarter	0.94 (0.91-0.97)		0.91 (0.90-0.93)		0.91 (0.89-0.93)
	Second quarter	0.94 (0.92-0.97)		0.91 (0.90-0.93)		0.91 (0.89-0.93)
	Third quarter	0.95 (0.93-0.97)		0.92 (0.91-0.93)		0.91 (0.90-0.93)
	Fourth quarter	0.95 (0.93-0.97)		0.89 (0.88-0.90)^h^		0.89 (0.87-0.91)

^a^DeLong test was used for comparing areas under the curve.

^b^*Z* test was used for comparing F1-scores.

^c^≤65 years versus >65 years; *P<*.001.

^d^Female versus male; *P<*.001.

^e^Surgery versus internal medicine or intensive care unit; *P<*.001; Bonferroni adjusted *P*=.003.

^f^The low, intermediate, and high risks of venous thromboembolism were defined by departments having <1, 1-5, and >5 venous thromboembolism–related deaths per year, respectively.

^g^Low versus high or intermediate; *P<*.001; Bonferroni adjusted *P*=.003.

^h^Fourth quarter versus third or second or first quarter; *P<*.001; Bonferroni adjusted *P*=.002.

**Table 7:** Error analysis of the clinical decision support system for venous thromboembolism risk assessment and integrated care for detecting venous thromboembolism.

Type of error, reasons for discrepancy		Values, n (%)
**False positive (n=55)**		
	Initially suspected to be venous thromboembolism but subsequently disproved	29 (53)
	Prior venous thromboembolism in the medical history but caught as on-admission venous thromboembolism	23 (42)
	The admission diagnosis was modified but did not get caught by the system	3 (5)
**False negative (n=63)**		
	Asymptomatic isolated calf muscle vein thrombosis occurred but not in the discharge diagnosis	44 (70)
	The admission diagnosis was modified but did not get caught by the system	12 (19)
	Unrecognized iliac vein thrombosis	7 (11)

### Subgroup Analysis

Subgroup analysis of the age, sex, and quarter of the study year demonstrated consistently good performance of the NLP tool in VTE detection with >87% sensitivity, specificity, PPV, and NPV, and >89% of AUC and F1-score. The NLP tool detected VTE in patients aged ≤65 years much better than that in patients aged >65 years (specificity 99.9% vs 99.5%; NPV 99.9% vs 99.5%; LR+ 1494 vs 178; F1-score 0.93 vs 0.89; respectively, all *P*<.001) (Table 5, Table 6, and Figure 4).

## Discussion

### Principal Findings

We developed an NLP algorithm with multisource EHR data and validated VTE detection extensively, in relation to different clinical settings with different VTE risks. The main findings of our study were as follows: (1) the NLP algorithm of DeVTEcare facilitated VTE detection with good diagnostic ability; (2) the NLP algorithm consistently detected the on-admission and in-hospital VTE well; however, the NLP algorithm showed higher sensitivity in detecting PE than DVT on admission; (3) among departments of surgery, internal medicine, and intensive care units, the highest specificity and NPV were shown in the surgery department; and (4) the NLP algorithm performed better among departments with low-risk VTE and among patients aged ≤65 years.

### Comparison With Prior Work

NLP can identify unstructured language that documents the diagnosis or findings of interest. Previous studies [10-13,16-24,31,32] on NLP embedded into EHRs to identify VTE were mainly based on imaging reports. Those studies reported about 94% sensitivity and 89% PPV for patients without surgery but only 81% sensitivity and 54% PPV for patients who had undergone a surgery (Multimedia Appendix 4) [10-13,16-24,31,32]. The NLP algorithm in our DeVTEcare not only confirmed good sensitivity, specificity, PPV, and NPV for VTE detection for patients admitted to internal medicine units but also for patients in the surgery department with higher specificity, NPV, and F1-scores compared to those for patients in the internal medicine and intensive care units.

Surgical procedures are associated with increased VTE risk, and certain surgeries, for example, orthopedic surgery, major general surgery, gynecological surgery, urological surgery, and neurosurgery are considered to pose a higher risk for VTE. Our algorithm for VTE detection was extensively validated in these clinical settings and was found to have good diagnostic ability. Our algorithm could help clinicians increase the awareness and prevention of VTE. The good performance of our NLP algorithm may be associated with the use of multisource data from EHRs besides imaging reports and the application of a set of highly optimized and configurable NLP components that enable rapid and straightforward clinical information processing. Moreover, the NLP algorithm of DeVTEcare demonstrated a better identification of on-admission PE than DVT, which was rarely reported by previous studies [19,20]. PE is a more severe clinical issue than DVT; nonetheless, it can be the preventable cause of hospital death. Thus, early identification of patients with PE on admission is critical. The adoption of adequate treatments in such patients during hospitalization would help to reduce adverse outcomes. Since the prevalence of VTE could vary across age, gender, season, and VTE risk, the performance of the NLP algorithm may be affected. However, our study showed that our NLP algorithm performed favorably in VTE identification across all subgroups. In addition, we found that the NLP algorithm detected VTE events even better for patients from departments at low VTE risk and those aged ≤65 years. This could be because VTE occurred less often in these patients, but the NLP system still recognized VTE well and thus showed better specificity while maintaining relatively high sensitivity. The NLP algorithm of DeVTEcare simplified and streamlined the screening of VTE for inpatients in different departments with different risks of VTE, and thus, it can be a user-friendly tool to facilitate the prevention of hospital-associated VTE and related morbidity.

Finally, the error analysis of our NLP tool for detecting VTE demonstrated that 81% of the false negative events were asymptomatic isolated calf muscle vein thrombosis and iliac vein thrombosis. This could partly explain the higher sensitivity of our NLP tool in PE detection than DVT detection on admission. In addition, 95% of the false positives were from patients with prior VTE or those with suspected VTE on admission but without confirmation of a diagnosis. The results from error analysis suggested that the text mining of key phrases needs to be enhanced furthermore.

### Limitations

There are several limitations in our study. First, although we tested the NLP algorithm extensively in different clinical settings, we cannot confirm if the findings of our study can be generalized to other hospitals, given the possible differences in the EHR structure, document formatting, and local terminologies. However, the rule-based NLP tool that employs a set of highly optimized and configurable NLP components can be easily tuned with a modest quantity of training data. Second, the VTE risk in the hospital departments was classified by the hospital quality control administration and we did not calculate the incidence rate of VTE, which is the standard procedure in epidemiology studies. Third, some physician-related factors such as the physician’s medical record writing style, awareness of VTE prevention and treatment, and imaging report quality have the potential to influence the NLP algorithm’s ability to identify VTE. Nonetheless, the hospital in our study is an academic teaching hospital in Beijing, which provides the highest quality of medical care in China. Fourth, given the retrospective design of this study, there was possible selection bias, which needs further external validation.

### Conclusions

The NLP algorithm of DeVTEcare identified VTE with good diagnostic ability across different clinical settings, especially for the surgery units, departments with low-risk VTE, and patients aged ≤65 years. This algorithm permits us to inform the accurate in-hospital VTE rates and enhance risk-classified VTE integrated care in future research.

## Appendix

Multimedia Appendix 1 Checklist of the Standards for Reporting Diagnostic accuracy studies (STARD) 2015 guidelines.

Multimedia Appendix 2 Keywords for the natural language processing algorithm in the clinical decision support system for venous thromboembolism risk assessment and integrated care.

Multimedia Appendix 3 Incidence of venous thromboembolism confirmed by clinical experts according to hospital inpatient department.

Multimedia Appendix 4 Summary of studies that identify venous thromboembolism using the natural language processing tool.

## Abbreviations

AUC: area under the receiver operating characteristic curve
DeVTEcare: clinical decision support system for venous thromboembolism risk assessment and integrated care
DVT: deep vein thrombosis
EHR: electronic health record
LR–: negative likelihood ratio
LR+: positive likelihood ratio
NLP: natural language processing
NPV: negative predictive value
PE: pulmonary embolism
PPV: positive predictive value
STARD: Standards for Reporting Diagnostic accuracy studies
VTE: venous thromboembolism

## Data Availability

The data that support the findings of this study are available from the corresponding author upon reasonable request.
