# Supplementary material for: Natural Language Processing in a Clinical Decision Support System for the Identification of Venous Thromboembolism: Algorithm Development and Validation
Source: J Med Internet Res. 2023 Apr 24;25:e43153. doi: 10.2196/43153 (PMC10167583; doi:10.2196/43153)
Supplement: Multimedia Appendix 2 [file jmir_v25i1e43153_app2.docx]

**Multimedia Appendix 2.** The keywords for the natural language processing algorithm in the DeVTEcare system.

|  | Pulmonary embolism | Deep vein thrombosis |
| --- | --- | --- |
| Keywords | 肺血栓栓塞\|肺栓塞\|肺动脉栓塞\|肺血栓\|栓塞\|肺动脉高压 | 肌\|下肢\|股\|腿\|胫\|腓\|腘\|股\|髂\|静脉血栓 |
| Exclusion keywords | 并发症为\|合并症为\|可出现\|检查\|引起\|导致\|危险因素\|高危\|中危\|低危\|避免\|谨防\|注意\|除外\|防止\|风险\|否认\|很少\|极少\|警惕\|考虑\|可能\|没有\|偶尔\|偶有\|排查\|排除\|少见\|是否\|未有\|未见\|未现\|无\|易现\|预防\|可导致\|可致\|未诉\|不伴\|今日未\|不充分\|易发生 | 浅静脉血栓\|并发症为\|合并症为\|可出现\|检查\|引起\|导致\|危险因素\|高危\|中危\|低危\|避免\|谨防\|注意\|除外\|防止\|风险\|否认\|很少\|极少\|警惕\|考虑\|可能\|没有\|偶尔\|偶有\|排查\|排除\|少见\|是否\|未有\|未见\|未现\|无\|易现\|预防\|可导致\|可致\|未诉\|不伴\|今日未\|不充分\|易发生 |
