# Supplementary material for: Natural Language Processing in a Clinical Decision Support System for the Identification of Venous Thromboembolism: Algorithm Development and Validation
Source: J Med Internet Res. 2023 Apr 24;25:e43153. doi: 10.2196/43153 (PMC10167583; doi:10.2196/43153)
Supplement: Multimedia Appendix 3 [file jmir_v25i1e43153_app3.docx]

**Multimedia Appendix 3.** The incidence of venous thromboembolism confirmed by clinical experts according to department.

| VTE risk  of department ^a^ | Department | PE ^b^  n (%) | DVT ^c^  n (%) | VTE ^d^  n (%) | Annual inpatients  (N) |
| --- | --- | --- | --- | --- | --- |
| High | Respiratory | 12 (1.5) | 33 (4.2) | 43 (5.5) | 783 |
|  | Neurology | 3 (0.3) | 39 (3.6) | 41 (3.7) | 1098 |
|  | Respiratory ICU | 7 (4.9) | 30 (21) | 35 (24) | 143 |
|  | Neurology ICU | 2 (1.4) | 21 (14) | 22 (15) | 147 |
|  | Medical Oncology | 4 (0.7) | 19 (3.3) | 21 (3.6) | 582 |
|  | Surgical ICU | 4 (3.9) | 18 (17) | 19 (19) | 102 |
|  | Nephrology | 0 (0) | 11 (2.2) | 11 (2.2) | 504 |
|  | Neurosurgery ICU | 1 (1.4) | 8 (12) | 9 (13) | 69 |
|  | Neurosurgery | 0 (0) | 6 (0.5) | 6 (0.5) | 1131 |
|  | TCM Orthopedic | 1 (0.2) | 4 (0.7) | 4 (0.7) | 582 |
|  | Special Duty Ward | 1 (0.2) | 2 (0.5) | 3 (0.7) | 408 |
|  | TCM Internal Medicine | 0 (0) | 2 (1.7) | 2 (1.7) | 115 |
|  | Interventional Radiology | 0 (0) | 1 (0.3) | 1 (0.3) | 391 |
|  | 1^st^ ward of Health Care | 1 (1.0) | 1 (1.0) | 1 (1.0) | 100 |
|  | Hepatobiliary and Pancreatic Surgery | 0 (0.0) | 1 (0.2) | 1 (0.2) | 614 |
|  | Sum (15 departments) | 36 (0.5) | 196 (2.9) | 219 (3.2) | 6769 |
| Intermediate | Cardiology | 93 (2.3) | 90 (2.2) | 133 (3.3) | 4027 |
|  | Rehabilitation | 3 (1.0) | 36 (11) | 39 (12) | 315 |
|  | General Surgery | 5 (0.3) | 25 (1.5) | 27 (1.6) | 1664 |
|  | Hyperbaric Oxygen Therapy | 2 (0.9) | 24 (11) | 26 (12) | 221 |
|  | Emergency | 3 (2.4) | 21 (17) | 24 (20) | 123 |
|  | Orthopedics | 1 (0.1) | 23 (2.1) | 23 (2.1) | 1075 |
|  | Gastroenterology | 3 (0.2) | 17 (0.9) | 18 (0.9) | 1907 |
|  | Gynecology | 3 (0.2) | 10 (0.8) | 13 (1.0) | 1256 |
|  | Gerontology | 2 (0.5) | 12 (2.8) | 13 (3.0) | 430 |
|  | Family Medicine | 0 (0) | 11 (2.4) | 11 (2.4) | 451 |
|  | Hematology | 0 (0) | 10 (5.3) | 10 (5.3) | 188 |
|  | Endocrinology | 3 (0.4) | 6 (0.9) | 8 (1.2) | 674 |
|  | Cardiac Surgery | 3 (0.8) | 4 (1.1) | 6 (1.7) | 362 |
|  | 2^nd^ ward of Health Care | 3 (1.7) | 5 (2.9) | 5 (2.9) | 172 |
|  | Thoracic Surgery | 2 (0.3) | 1 (0.1) | 2 (0.3) | 696 |
|  | Obstetric | 0 (0) | 0 (0) | 0 (0) | 1807 |
|  | Sum (16 departments) | 126 (0.8) | 295 (1.9) | 358 (2.3) | 15368 |
| Low | Cardiac Care Unit | 12 (4.0) | 13 (4.4) | 19 (6.4) | 298 |
|  | Ambulatory Care | 1 (0.1) | 18 (2.6) | 18 (2.6) | 684 |
|  | Urology | 2 (0.3) | 4 (0.7) | 6 (1.0) | 607 |
|  | Otomicrosurgery | 0 (0) | 3 (0.3) | 3 (0.3) | 1054 |
|  | Ophthalmology | 0 (0) | 2 (0.3) | 2 (0.3) | 788 |
|  | Otoscopic Surgery | 0 (0) | 1 (0.1) | 1 (0.1) | 1184 |
|  | Audiology Implantation | 0 (0) | 0 (0) | 0 (0) | 1135 |
|  | Pharyngeal and Voice Surgery | 0 (0) | 0 (0) | 0 (0) | 1472 |
|  | Nuclear Medicine | 0 (0) | 0 (0) | 0 (0) | 704 |
|  | Laminar Air Flow Room in Hematology Department | 0 (0) | 0 (0) | 0 (0) | 29 |
|  | TCM Acupuncture | 0 (0) | 0 (0) | 0 (0) | 49 |
|  | Pediatric ^e^ | 0 (0) | 0 (0) | 0 (0) | 11 |
|  | Sum (12 departments) | 15 (0.2) | 41 (0.5) | 49 (0.6) | 8015 |
| Total | 43 departments | 177 (0.6) | 532 (1.8) | 626 (2.1) | 30152 |

VTE: venous thromboembolism; PE: pulmonary embolism; DVT: deep vein thrombosis; ICU: Intensive Care Unit; TCM: traditional Chinese medicine

^a^ The low-, intermediate- and high-VTE risk of department was defined as having < 1, 1-5 and > 5 VTE-related death per year, respectively.

^b^ PE includes on-admission and in-hospital PE.

^c^ DVT includes on-admission and in-hospital DVT.

^d^ VTE includes on-admission and in-hospital VTE.

^e^ These adults hospitalized in paediatrics were prepared as bone marrow transplant providers.
