# Supplementary material for: Natural Language Processing in a Clinical Decision Support System for the Identification of Venous Thromboembolism: Algorithm Development and Validation
Source: J Med Internet Res. 2023 Apr 24;25:e43153. doi: 10.2196/43153 (PMC10167583; doi:10.2196/43153)
Supplement: Multimedia Appendix 4 [file jmir_v25i1e43153_app4.docx]

**Multimedia Appendix 4.** Summary of previous studies to identify venous thromboembolism using natural language processing tool.

| **Study** | **N** | **Patient type** | **Data type** | **NLP**  **approach** | **VTE type**  **(%)** | **Sensitivity**  **% (95% CI)** | **Specificity**  **% (95% CI)** | **PPV**  **% (95% CI)** | **NPV**  **% (95% CI)** |
| --- | --- | --- | --- | --- | --- | --- | --- | --- | --- |
| Verma *et al*. 2022 [10] | 4000/  PE: 500;  DVT: 445 | Medical  (40% from emergency) | Image | Symbolic | Patient-level:^a^  PE: 1.9  DVT: 1.2  R-level:^b^  PE: 9.0  DVT: 11.0 | Patient-level:  PE: 91.0 (NA)  DVT: 93.0 (NA)  R-level:  PE: 91.0 (NA)  DVT: 94.0 (NA) | Patient-level:  PE: 100 (NA)  DVT: 100 (NA)  R-level:  PE: 99.0 (NA)  DVT: 99.0 (NA) | Patient-level:  PE: 96.0 (NA)  DVT: 89.0 (NA)  R-level:  PE: 89.0 (NA)  DVT: 90.0 (NA) | Patient-level:  PE: 100 (NA)  DVT: 100 (NA)  R-level:  PE: 99.0 (NA)  DVT: 99.0 (NA) |
| Wendelboe, *et al*. 2022 [17] | NA/3078 | All | Image | Statistical | R-level:  VTE: 40.6 | R-level:  96.3 (96.2 - 96.4) | R-level:  92.0 (91.9 - 92.0) | R-level:  89.1 (89.0 - 89.2) | R-level:  97.3 (97.3 - 97.4) |
| Johnson *et al*. 2021 [11] | 960/960 | All | Image | Symbolic | R-level:  PE: 13 | R-level:  96.0 (90.9 - 98.7) | R-level:  97.7 (96.5 - 98.6) | R-level:  86.3 (79.5 - 91.6) | R-level:  99.4 (98.6 - 99.8) |
| Woller *et al*. 2021 [18] | 400/400 | All | Image | Symbolic | R-level:  PE: 8.7  DVT: 14.2 | R-level:  PE: 95.9 (89.4 - 99.5)  DVT: 96.1 (87.0 - 99.9)  VTE: 93.3 (82.9 - 99.1) | R-level:  PE: 99.1 (97.6 - 99.9)  DVT: 98.8 (97.7 - 99.6)  VTE: 99.6 (99.2 - 99.8) | R-level:  PE: 98.1 (94.7 - 99.8)  DVT: 95.1 (90.2 - 98.4)  VTE: 97.1 (94.3 - 98.9) | R-level:  PE: 98.0 (94.6 - 99.8)  DVT: 99.0 (96.5 - 100)  VTE: 99.0 (97.2 - 99.9) |
| Huesch *et al*. 2018 [19] | NA/1133 | Emergency | Image | Statistical | R-level:  PE: 7.6 | R-level:  100 (NA) | R-level:  97.0 (NA) | R-level:  73.3 (NA) | R-level:  100 (NA) |
| Dantes *et al*. 2018 [20] | 422/2083 | Surgical | Image | Statistical | P-level:  PE: 5.9  DVT: 4.0  VTE: 9.9  R-level:  PE: 3.3  DVT: 5.4  VTE: 8.7 | P-level:  PE:100 (86.2 - 100)  DVT: 94.1 (71.2- 99.0)  VTE: 97.6 (87.4 - 99.6)  R-level:  PE: 97.1 (89.9 - 99.6)  DVT: 97.3 (92.4- 99.4)  VTE: 97.2 (93.7 - 99.1) | P-level:  PE: 99.8 (98.6 - 100)  DVT: 100 (99.1- 100)  VTE: 99.8 (98.7 - 100)  R-level:  PE: 99.3 (98.5 - 99.7)  DVT: 99.4 (98.7- 99.8)  VTE: 99.3 (98.9 - 99.7) | NA | NA |
| Swartz *et al*. 2017 [21] | NA/2000 | All | Image | Symbolic | R-level:  PE: 14.0  DVT: 9.0 | R-level:  PE: 97.1 (94.3 - 99.9)  DVT: 95.7 (91.5 - 99.8) | R-level:  PE: 98.6 (97.8 - 99.4)  DVT: 100 (100 - 100) | R-level:  PE: 91.8 (87.3 - 96.2)  DVT: 100 (100 - 100) | R-level:  PE: 99.5 (99.1 -100 )  DVT: 99.6 (99.1 - 100) |
| Gálvez *et al*. 2017 [22] | 3371/6373 | Children | Image | Symbolic | Patient-level:  VTE: 3.0 | Patient-level:  82.9 (74.8 - 89.2) | Patient-level:  97.5 (96.9 - 98.0) | Patient-level:  54.5 (46.9 - 62.0) | Patient-level:  99.4 (99.0 - 99.6) |
| Tian *et al*. 2017 [23] | NA/1212 | All | Image | Symbolic | R-level:  PE: 12.2  DVT: 10.2 | R-level:  PE: 94.0 (89.0 - 97.0)  DVT: 94.0 (88.0 - 97.0) | R-level:  PE: 96.0 (95.0 - 97.0)  DVT: 96.0 (94.0 - 97.0) | R-level:  PE: 80.0 (73.0 - 85.0)  DVT: 73.0 (65.0 - 80.0) | R-level:  PE: 99.0 (98.0 - 100)  DVT: 96.0 (98.0 - 100) |
| Rochefort *et al*. 2015 [16] | 1751/2000 | All | Image | Statistical | R-level:  PE: 7.7  DVT: 16.2 | R-level:  PE: 79.0 (73.0 - 85.0)  DVT: 80.0 (76.0 - 85.0) | R-level:  PE: 99.0 (98.0 - 99.0)  DVT: 98.0 (97.0 - 99.0) | R-level:  PE: 84.0 (75.0 - 92.0)  DVT: 89.0 (85.0 - 93.0) | R-level:  PE: 98.0 (98.0 - 99.0)  DVT: 96.0 (93.0 - 99.0) |
| Shi *et al*. 2021 [12] | Internal  cohort: NA/2266  External  cohort: NA/16493 | Postoperative | EHR | Symbolic | Internal cohort  P-level :^c^  PE: 0.3  DVT: 0.6  External cohort  P-level :  PE: 0.4  DVT:1.0 | Internal cohort  P-level :  PE: 71.4 (28.6 - 100)  DVT: 84.6 (61.5 - 100)  External cohort  P-level :  PE: 81.5 (72.3 - 90.8)  DVT: 84.9 (79.1 - 90.1) | Internal cohort  P-level :  PE: 99.3 (98.9 - 99.6)  DVT: 99.3 (99.0 - 99.6)  External cohort  P-level :  PE: 99.8 (99.7 - 99.9)  DVT: 99.2 (99.1 - 99.4) | Internal cohort  P-level :  PE: 23.8 (12.5 - 40.0)  DVT: 42.3 (30.8 - 58.8)  External cohort  P-level :  PE: 63.2 (54.9 - 72.3)  DVT: 53.9 (49.6 - 58.9) | Internal cohort  P-level :  PE: 99.9 (99.8 - 100)  DVT: 99.9 (99.8 - 100)  External cohort  P-level :  PE: 99.9 (99.9 - 100)  DVT: 99.8 (99.8 - 99.9) |
| Selby *et al*. 2018 [24] | 703/757 | Postoperative | Image | Statistical | Patient-level:  PE: 4.3  DVT: 6.7 | Patient-level:  PE: 90.0 (NA)  DVT: 85.1 (NA) | Patient-level:  PE: 98.7 (NA)  DVT: 94.6 (NA) | Patient-level:  PE: 81.8 (NA)  DVT: 78.4 (NA) | Patient-level:  PE: 99.3 (NA)  DVT: 96.5 (NA) |
| FitzHenry *et al*. 2013 [31] | NA/4088 | Postoperative | EHR | Symbolic | P-level:  PE: 1.0  DVT: 2.0 | P-level:  PE: 80.0 (66.0 - 89.0)  DVT: 56.0 (45.0 - 67.0) | P-level:  PE: 97.0 (96.0 - 98.0)  DVT: 94.0 (93.0 - 95.0) | P-level:  PE: 23.0 (17.0 - 30.0)  DVT: 15.0 (11.0 - 20.0) | NA |
| Murff *et al*. 2011 [32] | 2327/NA | Postoperative | EHR | Symbolic | Patient-level:  PE: 0.7  DVT: 1.0 | Patient-level:  VTE: 59.0 (44.0 - 72.0) | Patient-level:  VTE: 91.0 (90.0 - 92.0) | NA | NA |

N: Number of validating patients/reports or procedures; NLP: natural language processing; VTE: venous thromboembolism; PE: pulmonary embolism; DVT: deep vein thrombosis; CI: confidence interval; NA: not applicable; PPV: positive predictive value; NPV: negative predictive value; R-level: Radiology report-level; P-level: Procedure-level; EHR: electronic health record.

^a^ Patient-level means that the data was derived from the analysis of each patient.

^b^ Radiology report-level means that the data was derived from the analysis of each radiology report.

^c^ Procedure-level means that the data was derived from the analysis of each operative event.
